# Protective Effect of Polyphenol-Rich Extract from Bee Pollen in a High-Fat Diet

**DOI:** 10.3390/molecules23040805

**Published:** 2018-03-31

**Authors:** Anna Rzepecka-Stojko, Agata Kabała-Dzik, Robert Kubina, Krzysztof Jasik, Maciej Kajor, Dorota Wrześniok, Jerzy Stojko

**Affiliations:** 1 Department of Pharmaceutical Chemistry, School of Pharmacy with the Division of Laboratory Medicine in Sosnowiec, Medical University of Silesia in Katowice, Jagiellonska 4, 41-200 Sosnowiec, Poland; dwrzesniok@sum.edu.pl; 2 Department of Pathology, School of Pharmacy with the Division of Laboratory Medicine in Sosnowiec, Medical University of Silesia in Katowice, Ostrogórska 30, 41-200 Sosnowiec, Poland; adzik@sum.edu.pl (A.K.-D.); rkubina@sum.edu.pl (R.K.); 3 Department of Skin Structural Studies, School of Pharmacy with the Division of Laboratory Medicine in Sosnowiec, Medical University of Silesia in Katowice, Kasztanowa 3, 41-200 Sosnowiec, Poland; kjasik@sum.edu.pl; 4 Department of Histopathology, School of Medicine in Katowice, Medical University of Silesia in Katowice, Medyków 18, 40-752 Katowice, Poland; katpat2@sum.edu.pl; 5 Department of Toxicology and Bioanalysis, School of Pharmacy with the Division of Laboratory Medicine in Sosnowiec, Medical University of Silesia in Katowice, Jagiellonska 4, 41-200 Sosnowiec, Poland; jstojko@sum.edu.pl

**Keywords:** bee pollen and polyphenols, lipid profile, liver, oxidised low density lipoproteins, asymmetric dimethylarginine, angiotensin-converting enzyme, angiotensin II

## Abstract

We have studied a preventive effect of polyphenol-rich bee pollen ethanol extract (EEP) against histological changes in the liver and cardiac blood vessels, abnormalities of lipid profile, and the levels of oxidized low density lipoproteins (ox-LDL), asymmetric dimethylarginine (ADMA), angiotensin-converting enzyme (ACE), and angiotensin II (ANG II) caused by a high-fat diet in C_57_BL_6_ mice. Supplementing the diet with EEP in the doses of 0.1 g/kg body mass (BM) and 1 g/kg BM resulted in a decrease of total cholesterol by 31% and 35%, respectively. It also decreased the level of low density lipoproteins by 67% and 90%, respectively. No differences in the levels of high density lipoprotein and triacylglycerols were observed. EEP reduced the level of ox-LDL by 33% and 47%, ADMA by 13% and 51%, ACE by 17% and 30%, as well as ANG II by 11% and 15% in a dose-dependent manner, which proves a protective effect of EEP in a high-fat diet. EEP reduces and/or prevents hepatic steatosis and degenerative changes caused by a high-fat diet in C_57_BL_6_ mice, which indicates its hepatoprotective effect. EEP used with standard feed does not disturb a normal concentration of the assayed parameters.

## 1. Introduction

Nowadays, there is a worldwide interest in natural products. They are used as dietary supplements in prophylaxis and treatment of many conditions, including lifestyle diseases such as atherosclerosis [1]. At present, the protection of human health involves mainly prophylaxis. Medical prophylaxis is a field of expertise whose activity aims not only at preventing the development of disease states but also at limiting the risk of their development [1,2].

Bee pollen is a natural bee product which belongs to apitherapeutics. It is characterised by a high antioxidant effect and various biological activities such as antibacterial ones (both, bactericidal and bacteriostatic activities), regenerative, detoxicative, and hepatoprotective activities. Moreover, it stimulates the immune system and activates cellular and systemic metabolisms [3,4,5,6]. Components of bee pollen, namely, amino acids, enzymes, bio-elements, vitamins, monosaccharides, and polysaccharides, and functional compounds (polyphenols) play a big role in the above-mentioned effects [7,8,9]. Polyphenols, chiefly phenolic acids and flavonoids, determine the antioxidant activity of bee pollen. They reduce the level of reactive oxygen species (ROS), ensure redox homeostasis, which results from interaction between pro- and antioxidant agents, and affect the improvement of physiological functions of the body. This has great importance for prophylaxis and treatment of conditions caused by oxidative stress [10,11,12,13].

Atherosclerosis is a chronic inflammation of the aorta and medium-sized arteries. Endothelial dysfunction is the key process that initiates the development of lesions in a vascular wall. Endothelial function can be impaired by increased oxidative stress, and can be manifested by abnormal vasodilation, increased macromolecule permeability, intensification of the inflammatory process and smooth muscle proliferation, and increased pro-thrombotic activity [14,15,16].

Disruption of vasodilator function of a vascular wall is related to limited production and bioavailability of nitrogen oxide (NO). Nitrogen oxide is produced by endothelial nitric oxide synthase (eNOS). It dilates endothelium and plays an important anti-inflammatory role, therefore it is an endothelial anti-atherosclerotic molecule [17,18]. Due to oxidative stress, excessive amounts of substances which inhibit eNOS and reduce NO bioavailability are produced in the body. They are, among others, asymmetric dimethylarginine [19,20], ox-LDL [14,21], angiotensin converting enzyme, and angiotensin II [22,23].

Taking into account the fact that bee pollen is a natural product of high antioxidant activity, the aim of this study was to determine the effect of polyphenol-rich bee pollen ethanol extract on changes in histopathological presentation of the liver and cardiac vessels, abnormalities in lipid profile, and the levels of ox-LDL, ADMA, angiotensin-converting enzyme (ACE), and ANG II due to a high-fat diet in C_57_BL_6_ mice. It was necessary to conduct the studies on the model which was not genetically determined for atherosclerosis in order to more fully estimate the protective effect of bee pollen ethanol extract (EEP) in hypercholesterolemia and atherosclerosis. These studies constitute also the reference for our research carried out on a standard animal model of atherosclerosis, i.e., C_57_BL_6_ ApoE-knockout mice [24]. To the best of our knowledge, the research reports on such studies cannot be found in available literature.

## 2. Results

### 2.1. Histopathological Tests of Liver

**Figure 1:**
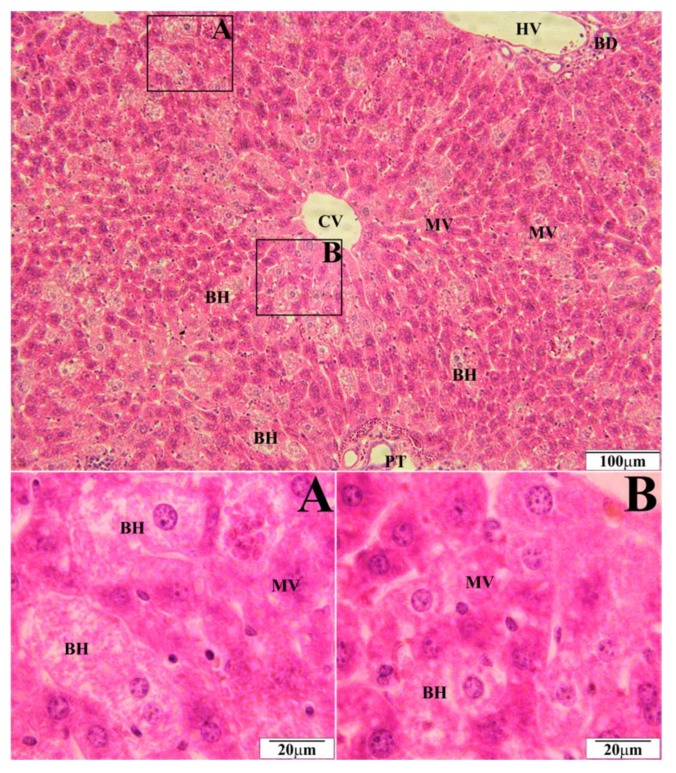
Representative section of the liver of C_57_BL_6_ mice on a high-fat diet (HFD). Slides stained with Hematoxylin and Eosin. BD—bile duct, BH—ballooned hepatocytes, CV—central vein, HV—hepatic portal vein, MV—microvesicular fatty hepatocytes, PT—portal triad.

**Figure 2:**
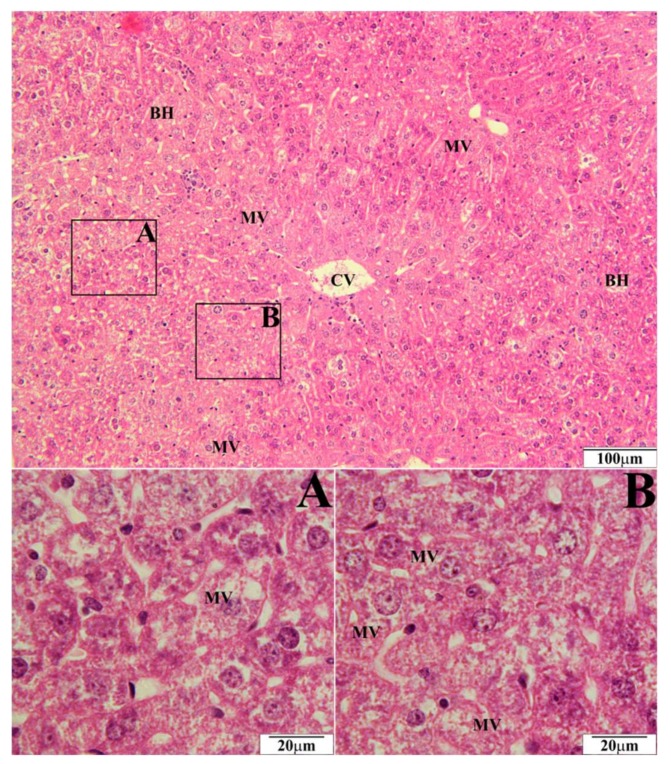
Representative section of the liver of C_57_BL_6_ mice on a high-fat diet supplemented with bee pollen ethanol extract (EEP) in the dose of 0.1 g/kg BM (HFD-0.1). Slides stained with Hematoxylin and Eosin. CV—central vein, BH—ballooned hepatocytes, MV—microvesicular fatty hepatocytes.

**Figure 3:**
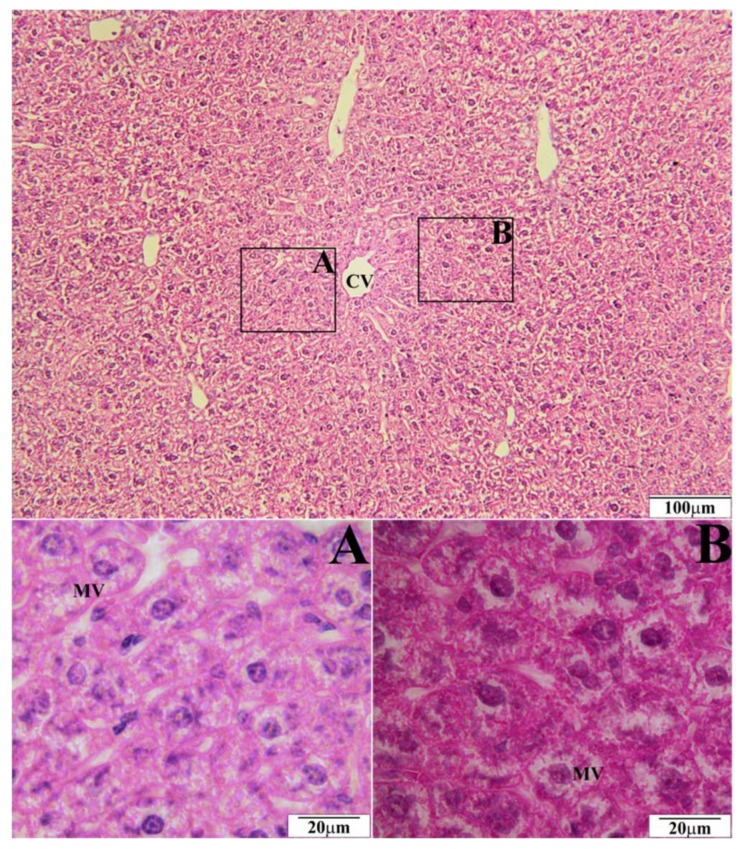
Representative section of the liver of C_57_BL_6_ mice on a high-fat diet supplemented with EEP in the dose of 1 g/kg BM (HFD-1). Slides stained with Hematoxylin and Eosin. CV—central vein, MV—microvesicular fatty hepatocytes.

In a histopathological tests of the liver, the biggest number of changes was observed in mice on a high-fat diet. Morphological changes were observed in the form of microvesicular fatty hepatocytes (Figure 1) whose intensity corresponded to 10 hepatocytes in the visual field. Simultaneously, ballooned hepatocytes were observed in the amount of over 20 hepatocytes in the visual field (Figure 1). In mice on a high-fat diet supplemented with EEP in the dose of 0.1 g/kg BM, microvesicular fatty hepatocytes were present in the same amount, i.e., up to 10 entities (Figure 2), whereas five ballooned hepatocytes were recorded in the visual field (Figure 2). In microscopic imaging of the livers of mice on a high-fat diet supplemented with EEP in the dose of 1 g/kg BM, 5 microvesicular fatty hepatocytes were observed, while no ballooned hepatocytes or other pathological changes were recorded (Figure 3). Histopathological presentation of the livers of mice on a standard diet supplemented with EEP in the doses of 0.1 g/kg BM and 1 g/kg BM, and without supplementation was similar. We only observed five microvesicular fatty hepatocytes in the visual field. 

### 2.2. Histopathological Tests of Arteries 

Histopathological evaluation of cardiac arteries was carried out in all experimental groups. Microscopic imaging did not reveal changes typical for the development of atherosclerosis in any group.

### 2.3. Effects of Ethanol Extract of Bee Pollen on the Lipid Profile 

The concentrations of total cholesterol (TC), triacylglycerols (TAG), high density lipoprotein (HDL cholesterol), and low density lipoprotein (LDL cholesterol) in the serum of C_57_BL_6_ mice in particular weeks of research within a given group did not reveal any statistically significant differences, and they were presented as mean values from the whole study period ± standard deviation. The results are presented in Table 1. TC and LDL cholesterol levels in particular experimental groups varied. The highest levels of the parameters were noted in mice on a high-fat diet. Supplementing a high-fat diet with EEP in doses of 0.1 g/kg BM and 1 g/kg BM resulted in a decrease of TC by 31% and 35%, respectively, as well as a decrease of LDL cholesterol level by 67% and 90%, respectively. Supplementing both, a high-fat diet and a standard diet with EEP, did not result in statistically significant differences in HDL level. On the other hand, there occurred statistically significant differences in HDL concentrations depending on the type of diet. Differences in TAG levels between particular experimental groups were not statistically significant.

### 2.4. Effect of EEP on Oxidized Low Density Lipoprotein 

**Figure 4:**
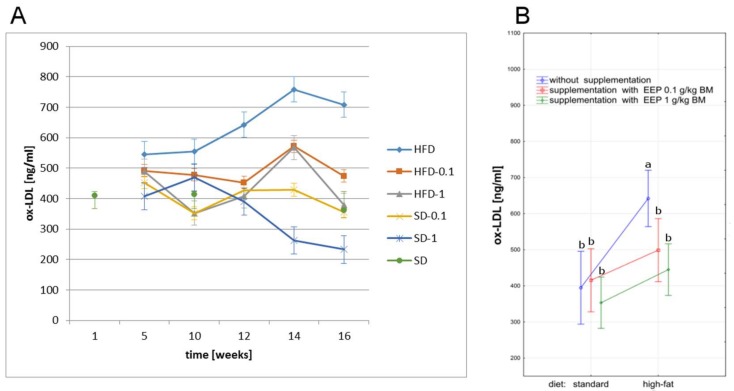
Oxidized low density lipoproteins level in C_57_BL_6_ mice. (**A**) Ox-LDL level in particular weeks of the study; (**B**) Average ox-LDL level (*n* = 10). HFD—high-fat diet; HFD-0.1—high-fat diet supplemented with EEP 0.1 g/kg BM; HFD-1—high-fat diet supplemented with EEP 1 g/kg BM; SD-0.1—standard diet supplemented with EEP 0.1 g/kg BM; SD-1—standard diet supplemented with EEP 1 g/kg BM; SD—standard diet. The highest concentration of ox-LDL was recorded in mice on a high-fat diet (HFD). In groups HFD-0.1 and HFD-1, the concentration of ox-LDL was much lower than in HFD. The lowest concentration of ox-LDL was recorded for a standard diet supplemented with EEP in the dose of 1 g/kg BM (SD-1); (*p* < 0.05; LSD ANOVA test; a—significant difference vs. SD, b—significant difference vs. HFD).

The highest concentration of ox-LDL in all study periods was recorded in mice on a high-fat diet. The high-fat diet led to an increase of ox-LDL up to 545 ng/mL, i.e., by 40% in the fifth week (Figure 4A), whereas in the 16th week ox-LDL increased to 709 ng/mL, i.e., by 79% (Figure 4A) when compared to an average concentration in the control group (395 ng/mL, Figure 4B). Supplementing a high-fat diet with bee pollen extract decreased ox-LDL level already in the fifth week, which was reduced to 474 ng/mL (Figure 4A) i.e., by 33% for the EEP dose of 0.1 g/kg BM, and to 378 ng/mL (Figure 4A) i.e., by 47% for the EEP dose of 1 g/kg BM (Figure 4A) in the 16th week. The lowest concentration of ox-LDL was recorded starting from the 12th week for a standard diet supplemented with EEP in the dose of 1 g/kg BM (Figure 4A).

### 2.5. Effect of EEP on *Asymmetric* Dimethylarginine

**Figure 5:**
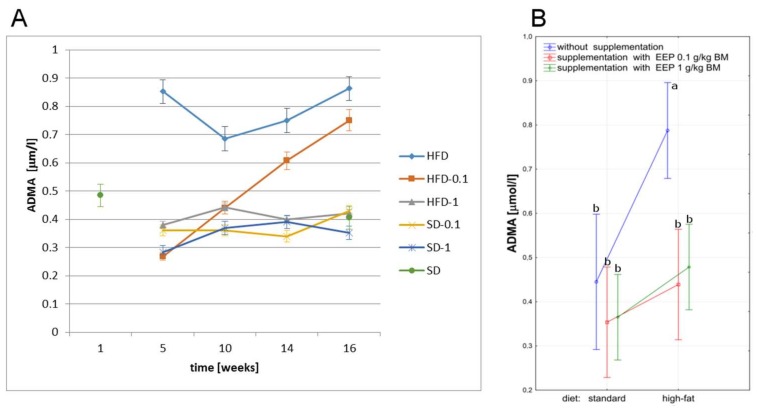
Asymmetric dimethylarginine level in C_57_BL_6_ mice. (**A**) ADMA level in particular weeks of the study; (**B**) Average ADMA level (*n* = 8). HFD—high-fat diet; HFD-0.1—high-fat diet supplemented with EEP 0.1 g/kg BM; HFD-1—high-fat diet supplemented with EEP 1 g/kg BM; SD-0.1—standard diet supplemented with EEP 0.1 g/kg BM; SD-1—standard diet supplemented with EEP 1 g/kg BM; SD—standard diet. ADMA levels in mice on a high-fat diet supplemented with EEP in the dose of 1 g/kg BM (HFD-1) were similar to the parameter level recorded for a standard diet (SD); (*p* < 0.05; LSD ANOVA test; a—significant difference vs. SD, b—significant difference vs. HFD).

The analysis of the effect of a high-fat diet on ADMA level showed that such feeding caused an increase of this parameter value during the whole study period, and the difference was statistically significant when compared to all other groups (Figure 5B). Already in the fifth week, a high-fat diet resulted in an increase of ADMA level to 0.852 μmol/L (Figure 5A) i.e., by 91%, and to 0.863 μmol/L (Figure 5A) i.e., by 93% in the 16th week, when compared to an average level in the control group (0.445 μmol/L, Figure 5B). Supplementing a high-fat diet with EEP in both doses reduced ADMA level during the whole study period, and the difference was statistically significant (Figure 5B). The supplementation with EEP for 16 weeks in the dose of 0.1 g/kg BM resulted in a decrease of ADMA level to 0.750 μmol/L (Figure 5A) i.e., by 13%, and to 0.420 μmol/L (Figure 5A) i.e., by 51% in the dose of 1 g/kg BM, when compared to an unsupplemented high-fat diet. ADMA levels in mice on a high-fat diet supplemented with EEP in the dose of 1 g/kg BM during the whole study period were similar to the parameter level recorded for a standard diet supplemented with EEP both, in the dose of 1 g/kg BM and 0.1 g/kg BM (Figure 5A,B).

### 2.6. Effect of EEP on Angiotensin-Converting Enzyme

**Figure 6:**
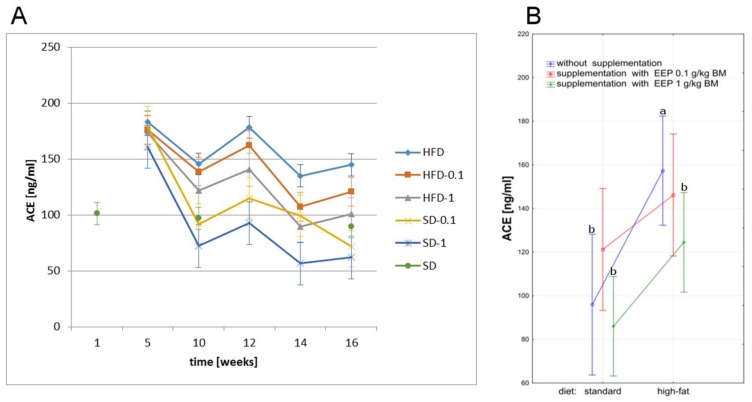
Angiotensin-converting enzyme level in C_57_BL_6_ mice. (**A**) Aangiotensin-converting enzyme (ACE) level in particular weeks of the study; (**B**) Average ACE levels (*n* = 10). HFD—high-fat diet; HFD-0.1—high-fat diet supplemented with EEP 0.1 g/kg BM; HFD-1—high-fat diet supplemented with EEP 1 g/kg BM; SD-0.1—standard diet supplemented with EEP 0.1 g/kg BM; SD-1—standard diet supplemented with EEP 1 g/kg BM; SD—standard diet. In HFD-1 group, the concentration of ACE was much lower than in HFD; (*p* < 0.05; LSD ANOVA test; a—significant difference vs. SD, b—significant difference vs. HFD).

A high-fat diet has a big influence on variations in ACE level, which is revealed by a statistically significant increase of this parameter when compared to the other groups (Figure 6B) A high-fat diet led to an increase in ACE level already in the fifth week, which was 145 ng/mL (Figure 6A) in the 16th week, and by 51% higher when compared to an average level in the control group (96 ng/mL, Figure 6B). Supplementing a high-fat diet with EEP resulted in lowering this parameter level to 121 ng/mL (Figure 6A) i.e., by 17% after 16 weeks when the dose was 0.1 g/kg BM, and to 101 ng/mL (Figure 6A) i.e., by 30% for the dose of 1 g/kg BM when compared to the unsupplemented group. The lowest values of the parameter were recorded for a standard diet supplemented with EEP in the dose of 1 g/kg BM, however, the differences were not statistically significant when compared to the unsupplemented group (Figure 6A,B).

### 2.7. Effect of EEP on Angiotensin II

**Figure 7:**
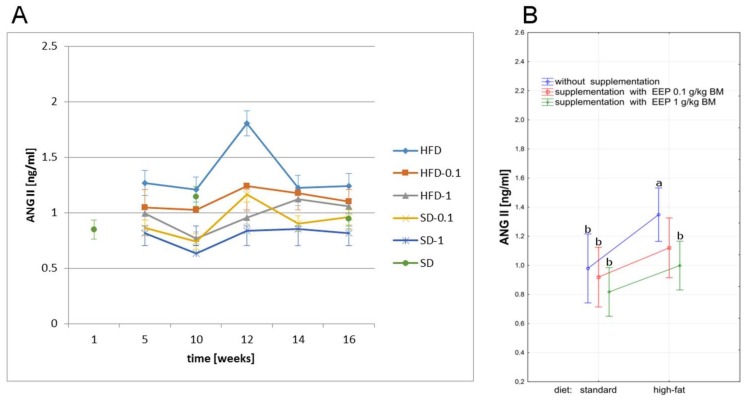
Angiotensin II level in C_57_BL_6_ mice. (**A**) ANG II level in particular weeks of the study; (**B**) Average ANG II level (*n* = 10). HFD—high-fat diet; HFD-0.1—high-fat diet supplemented with EEP 0.1 g/kg BM; HFD-1—high-fat diet supplemented with EEP 1 g/kg BM; SD-0.1—standard diet supplemented with EEP 0.1 g/kg BM; SD-1—standard diet supplemented with EEP 1 g/kg BM; SD—standard diet. The highest ANG II concentration was recorded in HFD. In HFD-1 group, during the total period of observation, lower ANG II concentration was recorded when compared to HFD group; (*p* < 0.05; LSD ANOVA test; a—significant difference vs. SD, b—significant difference vs. HFD).

A high-fat diet caused an increase of ANG II during the whole study period when compared to all other groups. It led to an increase of ANG II level already in the fifth week, and the highest value was recorded in the 12th week, whereas it was 1.24 ng/mL (Figure 7A) in the 16th week, and was by 27% higher than an average level in the control group (0.98 ng/mL, Figure 7B). Supplementing a high-fat diet with EEP for 16 weeks resulted in a decrease of ANG II to 1.10 ng/mL i.e., by 11% when the dose was 0.1 g/kg BM, and to 1.06 ng/mL (Figure 7A) i.e., by 15% for the dose of 1 g/kg BM (Figure 7A) when compared to an unsupplemented high-fat diet. Supplementing a high-fat diet with bee pollen extract in the dose of 1 g/kg BM caused a statistically significant decrease of ANG II level during the total study period. The lowest ANG II levels were observed in mice fed with a standard feed supplemented with EEP in the dose of 1 g/kg BM, however the differences were not statistically significant when compared to the unsupplemented group (Figure 7A,B).

## 3. Discussion

An appropriate diet that provides natural antioxidants is a very important element of the prophylaxis of atherosclerosis, cardiovascular diseases, and many other conditions caused by oxidative stress [25,26].

Bee pollen is a natural product which is characterised by a high efficiency of ROS neutralisation, and consequently by a high biological activity. These characteristics result from the presence of strong antioxidants, namely polyphenols, in its composition [27,28,29]. The mechanism of antioxidant activity of polyphenols involves the following: inhibiting the activity of enzymes which catalyse ROS; chelating metal ions involved in the formation of free radicals; scavenging of free radicals which counteracts lipid peroxidation; and synergistic action with other antioxidants, e.g., vitamin C and tocopherols. Epidemiological, clinical, and dietary studies indicate that populations whose diet is rich in polyphenols are less susceptible to cardiovascular diseases and complications related to them [13,30,31].

In this presented research, we evaluated a preventive effect of bee pollen ethanol extract against changes in histopathological presentation of the liver and cardiac vessels, abnormalities of lipid profile, and the levels of ox-LDL, ADMA, ACE, and ANG II caused by a high-fat diet in C_57_BL_6_ mice. The studies were conducted on the model which was not genetically determined for atherosclerosis. This was necessary in order to obtain a more complete evaluation of a protective effect of EEP against hypercholesterolemia and atherosclerosis. The studies constitute a reference for our research carried out on a standard experimental model of atherosclerosis, i.e., C_57_BL_6_ ApoE-knockout mice [24].

A high-fat diet is one of the factors that lead to disorders of lipid metabolism. The liver is the organ that plays the main role in lipid transformations. Therefore, providing big amounts of fats in the diet can cause liver dysfunction and development of steatosis and inflammation. Coexistence of steatosis and balloon disease (inflammation with damage to hepatocytes) referred to as non-alcoholic steatohepatitis (NASH) can lead to cirrhosis and liver failure, and sometimes to liver cancer [32].

To our best knowledge, we have been the first ones to prove that EEP has a hepatoprotective effect on C_57_BL_6_ mice on a high-fat diet. Supplementing a high-fat diet with bee pollen ethanol extract both, in the dose of 1 g/kg BM and 0.1 g/kg BM, has significantly reduced or prevented the development of ballooned hepatocytes in the liver (Figure 2 and Figure 3). On the other hand, such numerous changes were present when a high-fat diet was not supplemented (Figure 1). A hepatoprotective effect is related to a significant decrease of TC level (by 31% and 35%; Table 1), and LDL cholesterol level (by 67% and 90%; Table 1). This decrease was obtained due to supplementing a high-fat diet with EEP. 

The published data show that supplementation with different polyphenols reduced the inflammatory profile in the serum/liver induced by a high fat diet contributing to the amelioration of fatty liver dysfunction and its progression to NASH. The mechanisms underlying such observations are likely to include improved adipokine regulation and insulin sensitivity, a decline in de novo lipogenesis (via sterol regulatory element-binding protein 1c; SREBP-1c) and an increase in fatty acids β-oxidation activity which would reduce the lipid load in the liver [33].

We showed the normalisation of TC level due to EEP supplementation in our research on C_57_BL_6_ ApoE-knockout mice, which are a standard experimental model of atherosclerosis [24]. Lipid metabolism is also normalised by propolis, which, like bee pollen, belongs to a group of bee products [34]. Propolis extract also limits atherosclerotic changes in cardiac vessels [35,36].

According to literature data, the modulation of lipid levels with polyphenols can take place through various mechanisms. Flavonoids reduce hypercholesterolemia by inhibiting hepatic synthesis of apolipoprotein B, which is a part of the VLDL and LDL lipoproteins [37]. They reduce COX2 synthesis, inhibit the release of prostaglandin E2 and reduce gene expression for pro-inflammatory cytokines such as IL1α, IL1β IL6, and TNFα in macrophage cells [38]. In addition, they inhibit the cellular absorption of VLDL, block the SR-A receptor (membrane receptor responsible for accumulation of cholesterol esters in macrophages and foam cell formation) and impede the metabolism of acylated LDL by macrophages as well as inhibit the transformation of macrophages into foam cells in the vascular wall [39]. They reduce the expression of the HNF4α, gene which affects the secretion of apolipoprotein B and the metabolism of glucose and lipids [40]. The hypolipidemic effect of polyphenols is also associated with the reduction of the acyl-CoA: cholesterol acyltransferase (ACAT) activity involved in the metabolism of VLDL [41].

Ox-LDL molecules constitute an important factor initiating the development of atherosclerosis. The molecules are formed in the process of oxidation of low density lipoproteins under the influence of oxidative stress [42]. Ox-LDL, acting through specific endothelial receptors, activates the endothelium for the production of pro-inflammatory cytokines, chemokines, and enhances the expression of adhesion proteins, which causes the activation of monocytes and their differentiation into macrophages, and stimulation of lymphocytes. This leads to the destabilisation of the vascular wall and further changes [43]. Consequently, the uncontrolled absorption of ox-LDL by macrophages and the transformation of macrophages into foam cells is increasing [44]. In the inner membrane of the blood vessel, a fatty streak is formed, which consists of foam cells, macrophages, and T lymphocytes. Continuous accumulation of lipoproteins as well as proliferation and migration of smooth muscle cells causes the formation and development of atherosclerotic plaque [15].

The LDL oxidation process can be reduced by introducing antioxidants such as α- and γ-tocopherol, carotenoids, ascorbic acid, lycopene, and polyphenols into the diet [45]. The literature suggests that polyphenols of different botanical origins reduce oxidative stress, thus they are inhibitors of oxidative modification of LDL lipoproteins. They improve the function of the endothelium, therefore they play an important role in the prevention of atherosclerosis [11,46,47]. This effect is associated with increased activity of endothelial nitric oxide synthase, reduction of hydrogen peroxide, leucotriene B4, and concentration of P-selectin [48]. Polyphenols limit the expression of the inflammatory factor NFκB at transcription level, and reduce the level of metalloproteinase 9 [12]. They attenuate the expression of adhesion molecules of VCAM1 and ICAM1 cells [49]. Moreover, they reduce the level of monocytes and lymphocytes [50].

Our studies lead to a conclusion that supplementing a high-fat diet with bee pollen ethanol extract for 16 weeks causes a decrease of ox-LDL in mice both, in the dose of 1 g/kg BM and 0.1 g/kg BM, by 33% and 47%, respectively (Figure 4A). A high-fat diet has a huge impact on variations in ox-LDL level, which was manifested by a high concentration of this parameter, and the difference was statistically significant when compared to other groups (*p* < 0.05, Figure 4B). Supplementing a standard diet with EEP irrespectively of the dose, did not lead to statistically significant differences in the concentration of the parameter. The lowest levels of ox-LDL were recorded for long-lasting supplementation of a standard diet with EEP in the dose of 1 g/kg BM.

We showed that polyphenol fraction of bee pollen significantly decreases a high level of ox-LDL caused by a high-fat diet in C_57_BL_6_ mice. This is related to a big content of polyphenols and a high antioxidant activity of EEP resulting from it. Inhibiting lipid peroxidation proves a high efficiency of bee pollen ethanol extract in reducing oxidative stress. Therefore, EEP can be used in prophylaxis of atherosclerosis, cardiovascular diseases and other conditions caused by oxidative stress.

Endothelial dysfunction observed in various diseases is a consequence of, among other things, high concentrations of asymmetric dimethylarginine. Asymmetric dimethylarginine is an endogenous inhibitor of nitric oxide synthase. ADMA participates in one of the mechanisms limiting the bioavailability of nitric oxide, which is an endogenous substance with a strong anti-atherosclerotic effect [17,51]. Metabolism is mediated by dimethylarginine-dimethylamine hydrolase (DDAH) and leads to the breakdown of ADMA to citrulline and dimethylamine [52]. The reduction of DDAH activity occurs under the influence of oxidative stress, inflammation and hypercholesterolemia [53]. The increase in ADMA concentration reduces the synthesis of NO, and causes damage to the endothelial function, increases oxidative stress and monocyte adhesion. Increased ADMA levels are observed in people with kidney disease, atherosclerosis, diabetes, hypertension, hypercholesterolaemia, hyperhomocysteinaemia, as well as in tobacco smokers [20,54,55,56]. Consequently, this allows for identification of ADMA as an early marker of biochemical endothelial dysfunction in the case of atherosclerosis, cardiovascular disease, and insulin resistance [17,20,22,51].

According to literature, ADMA level increases in the serum of hypercholesterolaemic rabbits due to cholesterol-enriched diets. This indicator, in experimental animals, changes at a very early stage of development of atherosclerotic lesions [51]. ADMA levels are also rapidly increasing in people with endothelial dysfunction. Clinical and experimental studies suggest that an increase in ADMA is a prodromal symptom and occurs earlier than the clinical symptoms of cardiovascular disease [57,58].

In our studies, we observed a similar trend of changes of this parameter. ADMA level in C_57_BL_6_ mice on a high-fat diet was the highest one when compared to the other groups during the total observational period. Our research also suggests that supplementing a high-fat diet with bee pollen ethanol extract lowers ADMA level to the level determined for a standard diet. It is very important for prevention of atherosclerosis since EEP reduces endothelial dysfunction due to an increase of NO bioavailability. Our research has been the first one to prove the effect of bee pollen ethanol extract on the level of ADMA, i.e., a potential biomarker of endothelial dysfunction. Based on the presented studies, it can be stated that bee pollen ethanol extract can be useful for the prophylaxis of atherosclerosis.

The literature shows a similar effect with olive oil because it contains a large amount of polyphenols. Its consumption causes a significant reduction in the levels of ADMA, ox-LDL, and CRP acute phase protein in young women with slightly elevated or over-positive blood pressure [56]. In diabetic mice, silibinin, a polyphenolic compound contained in silymarin, lowers insulin resistance and reduces endothelial dysfunction as a result of decreased ADMA levels [55].

Oxidative stress contributes to the increase in the activity of angitensin-converting enzyme and the increase in the synthesis of angiotensin II. Angiotensin convertase can cause adverse structural and functional changes in the endothelium, and interfere with the renin-angiotensin-aldosterone system homeostasis (RAA) by lowering bradykinin concentration, reducing the release of endothelial nitric oxide, prostacyclin (PGI2) and tissue plasminogen activator (t-PA). It also causes an increase in the concentration of angiotensin II, which is an important atherogenic agent [59,60].

Angiotensin II affects oxidative stress and atherosclerosis. Angiotensin II may directly or indirectly induce endothelial dysfunction, especially in the function of nitric oxide concentration. The adverse effect of angiotensin II on vasodilator functions of endothelium may result from stimulation of ROS production, which contributes to the formation of ox-LDL [21,61].

In our research, we have presented the effect of supplementing a high-fat diet with bee pollen ethanol extract on the levels of ACE and ANG II in C_57_BL_6_ mice. Supplementing a high-fat diet with EEP in the doses of 0.1 g/kg BM and 1 g/kg BM for 16 weeks results in lowering the concentration of ANG II by 11% and 15%, respectively (Figure 7A) when compared to an unsupplemented high-fat diet. This is related to lowering the level of angiotensin-converting enzyme by 17% when the dose was 0.1 g/kg BM, and by 30% for 1 g/kg BM (Figure 6A). Based on the study results, it can be suggested that polyphenol fracture from bee pollen, due to a high antioxidant activity, improves endothelial functions by modulation of the renin-angiotensin-aldosterone system and may play a protective role in cardiovascular diseases.

The literature data show that polyphenols weaken ACE activity by lowering the level of ROS, which is the result of their antioxidant properties [62,63]. Genistein, a flavonoid from the isoflavone group, stimulates the synthesis of nitric oxide in endothelial cells, lowers the expression and activity of ACE in the endothelium and in the serum of rats [64]. Taxifolin inhibits ACE activity in the aorta of rats [62]. Quercetin has a protective effect on vessels in mice deficient in apolipoprotein E [65]. In rats with spontaneous hypertension, quercetin lowers blood pressure and eliminates pathological changes in the vessels [66]. Anti-hypertensive action of polyphenols is associated with eNOS activation and inhibition of metalloproteinase-2 [67], and modulation of the renin-angiotensin-aldosterone system as a result of the reduction of antioxidative stress [68]. In vitro, bee pollen extract also inhibits ACE activity, which is the result of high antioxidant potential [69].

## 4. Materials and Methods 

### 4.1. Preparation of Ethanol Extract of Bee Pollen 

The material for the tests were ground pollen loads obtained from ecologically clean harvest areas in the south of Poland. Samples of polyfloral bee pollen were collected from an apiary at Kamianna (GPS N 49°31′527, E 20°56′116), Poland, in the Beskidy Mountains. From May to July, bee pollen samples were collected by beekeepers with the use of pollen traps mounted on selected beehives. Ethanol extract was prepared according to a slightly modified method of Almaraz-Abarca et al. [27]. The ethanol extract of bee pollen was prepared by weighing 20 g of ground bee pollen, with accuracy of 0.01 g. Then the bee pollen sample was extracted 5 times with 50% (*v*/*v*) ethanol aqueous solution, in 200 mL portions, and shaken each time for 60 min at room temperature, in order to macerate the sample. After each extraction, the sample was filtered under reduced pressure with the use of a water pump. The filtrate was collected, and substrate was extracted again with another portion of ethanol. The obtained filtrate was centrifuged at 10,000 rpm for 10 min, and then it was evaporated under reduced pressure in a rotary vacuum evaporator (UNIPAN-PRO 350P). The evaporated extract was dried in a laboratory incubator at 38 °C to obtain solid mass. 

Prior to in vivo studies, bee pollen ethanol extract was analysed for total content of polyphenols and flavonoids as well as for antioxidant activity. The content of particular flavonoids in EEP, which were identified and determined with the use of HPLC method, was as follows: rutin 5.483 mg/g; myricetin 2.657 mg/g; quercetin 1.350 mg/g; isorhamnetin 1.054 mg/g; kaempferol 0.724 mg/g. Phenolic acids present in EEP are: gallic acid 3.922 mg/g; trans-cinnamic acid 1.269 mg/g; 4-hydroxycinnaminic acid 0.898 mg/g; felluric acid 0.819 mg/g; 4-trans-p-coumaric acid 0.584 mg/g; caffeic acid 0.058 mg/g. Total polyphenol content was 27 mg GAE/g, and flavonoids constituted 20 mg QE/g. EEP had high antioxidant activity, which was determined based on the reduction of DPPH (EC_50_ = 57.5 μmol/g) and ABTS^•+^ (TEAC = 0.692 mmol/g) [28]. 

### 4.2. Animals and Treatments 

The study was conducted on 56 females of C_57_BL_6_ mice, aged 4 weeks, 25 ± 5 g of body mass. During the experiment, the animals were kept in standard breeding conditions, i.e., in groups of 10 animals in polypropylene cages, in rooms of constant temperature (23 ± 2 °C), and constant air humidity (50–70%), keeping the daytime rhythm in the inflow of light (a 12-h cycle). 

The high-fat diet (HFD) comprised High Fat Rodent Diet supplemented with 21% lard and 0.15% cholesterol (Special Diets Services, Witham, Essex, UK). The standard diet (SD) was standard feed (Labofeed B) containing 17% protein, 3.5% fat and 38% carbohydrates (Animal Feed Manufacturer “Morawski” in Kcynia). Ethanol extract of bee pollen was added to feed in a dose of 0.1 and 1 g/kg BM, respectively. Two dosing levels of EEP used in the research were calculated based on the daily ingestion of polyphenols in the diet of the inhabitants of Central Europe [10].

Experimental animals were divided into 6 groups according to the following scheme: study groups: HFD; HFD-0.1; HFD-1; SD-0.1; and SD-1 (10 animals in each group), and a control group SD (six animals). Characteristics of the groups: HFD—high-fat diet, HFD-0.1—high-fat diet supplemented with EEP (0.1 g/kg BM), HFD-1—high-fat diet supplemented with EEP (1 g/kg BM), SD-0.1—standard diet supplemented with EEP (0.1 g/kg BM), SD-1—standard diet supplemented with EEP (1 g/kg BM), and SD—standard diet. 

The project with the use of animal models was approved by the Local Ethics Committee on Animal Experimentation of the Medical University of Silesia in Katowice.

### 4.3. Collecting Biological Material for Tests 

The material for tests was collected in the fasted state, in the fifth, 10th, 12th, 14th, and 16th week of the experiment, after general anaesthesia with a drug called Tiopental (Sandoz, Poland) in a dose of 20 mg/kg BM, administered by injection. 

Blood for analyses was collected by puncturing the tip of the heart with a cannula. Blood was collected to chemically pure test tubes in order to obtain serum. Blood after clotting and clot retraction was centrifuged, and serum was frozen at −80 °C, and stored for further tests. 

The collection of material for histopathological tests—before the material was collected, perfusion was performed by decompression surgery in the 1/3 of descending abdominal aorta. Perfusion was performed with phosphate buffered saline (PBS) until the light pink translucent liquid flew out in the decompressing orifice. Then, perfusion with 10% PBS-buffered formaldehyde was performed for 2 min to obtain preliminary fixing of the heart and trunks of major blood vessels. Next, the liver, the heart and the brachiocephalic trunk were fixed in 10% PBS-buffered formaldehyde, and kept for further tests. Acetone, PBS, formalin were purchased from POCh (Gliwice, Poland).

### 4.4. Histopathological Tests 

Histopathological evaluation of the liver, the heart and the brachiocephalic trunk was conducted in the 16th week of the study. The collected material was fixed in a 10% formalin solution for minimum 24 h. After fixing, the tissues were treated with the aqueous solutions of ethyl alcohol. The material was subsequently rinsed with acetone and xylene, respectively. Then, tissues were placed in a xylene and paraffin mixture at a 1:1 ratio and then in liquid paraffin. After solidification, the paraffin was cut into 3–6 micron thick bands. Paraffin bands were treated with xylene, an ethyl alcohol/xylene mixture at a 1:1 ratio and aqueous solutions of ethyl alcohol, and then rinsed in distilled water. The material prepared in this way was stained with the standard hematoxylin-eosin (HE) method, which included staining with alkaline solution of hematoxylin, rinsing in distilled water, staining with acid solution of eosin, and rinsing in distilled water. Later, preparations were treated with aqueous solutions of ethyl alcohol, ethanol/xylene mixture at 1:1 ratio and rinsed in xylene in order to finally dehydrate and radiate the tissue. Slides were closed with cover glass by means of DPX-medium. Xylene, hematoxylin, eosin aqueous solution (1%), anhydrous ethyl alcohol (99.8%), acetone, PBS, formalin were purchased from POCh (Gliwice, Poland). DPX mounting medium was purchased from Fluka (Dresden, Germany).

Histopathological preparations were evaluated in 40×, 100×, and 200× magnifications using Olympus BX60 microscope equipped with XC50 digital camera and Olympus cellSens Standard software (Olympus Corp., Tokyo, Japan).

### 4.5. Biochemical Tests 

We tested serum obtained in the scheduled study periods, i.e., in the fifth, 10th, 12th, 14th, and 16th week of the experiment for the study groups HFD, HFD-0.1, HFD-1, SD-0.1, SD-1, and in the 1st, 10th, and 16th week for the control group (SD). The levels of ox-LDL, ADMA, ACE, and ANG II were determined with Asys HiTech UVM 340 microplate reader and Micro Win 4.35.

#### 4.5.1. Serum Lipid Profile Analysis 

Total cholesterol, triacylglycerols, high density lipoprotein and low density lipoprotein serum level were determined with an enzymatic method. A reagent set from Beckman Coulter (Praha, The Czech Republic) was used for the determination, which was conducted according to the manufacturer’s instruction for Beckman Coulter AU 680 analyzer. TC, TAG, HDL cholesterol, and LDL cholesterol levels were given in mg/dL.

#### 4.5.2. Determination of Oxidized Low Density Lipoprotein Concentration 

The concentration of ox-LDL was determined with a sandwich ELISA using Mouse Oxidized Low Density Lipoprotein ELISA Kit (Cat. No. E90527Mu was purchased from Uscn Life Science Inc., Wuhan, China). The analysis was conducted according to the manufacturer’s instruction. 

The microtiter plate was pre-coated with an antibody specific to ox-LDL. Samples diluted 10 times or standards were then added to the appropriate microtiter plate wells, and incubated (for 2 h at 37 °C). Then, the liquid was removed and a biotin-conjugated polyclonal antibody specific for ox-LDL was added. The plate was incubated (for 1 h at 37 °C). Then the plate was washed, and avidin conjugated to horseradish peroxidase (HRP) was added to each microplate well, and the plate was incubated (for 1 h at 37 °C). Next, a 3,3′,5,5′-tetramethyl-benzidine (TMB) substrate solution was added to each well and incubated (for 30 min at 37 °C). Only those wells that contained ox-LDL, biotin-conjugated antibody and enzyme-conjugated avidin would exhibit a change in colour. The enzyme-substrate reaction was terminated by the addition of a sulphuric acid solution, and the colour change was measured spectrophotometrically at a wavelength of 450 nm. The concentration of ox-LDL (ng/mL) in the samples was then determined by comparing the O.D. of the samples to the standard curve. 

#### 4.5.3. Determination of Asymmetric Dimethylarginine Concentration

The concentration of asymmetric dimethylarginine was determined with ADMA ELISA Kit for the determination of ADMA (Cat. No. K 3001 was purchased from Immundiagnostik AG, Bensheim, Germany). The assay was conducted according to the manufacturer’s instruction. 

This assay is based on the method of competitive enzyme-linked immunoassays. The sample preparation includes the addition of a derivatization-reagent for ADMA coupling. Afterwards, the treated samples and the polyclonal ADMA-antiserum are incubated in wells of microplate coated with ADMA-derivative (tracer) for 18 h at 4–8 °C. During the incubation period, the target ADMA in the sample competes with the tracer immobilised on the wall of the microtiter wells for the binding of the polyclonal antibodies. The ADMA in the sample displaces the antibodies out of the binding to the tracer. Therefore the concentration of the tracer-bound antibody is inversely proportional to the ADMA concentration in the sample. During the second incubation step (for 1 h at 18–26 °C), a peroxidase-conjugated antibody is added to each microtiter well to detect the anti-ADMA antibodies. After washing away the unbound components, 3,3′,5,5′-tetramethyl-benzidine is added as a substrate for peroxidase and then incubated (for 10 min at 18–26 °C). Finally, the enzymatic reaction is terminated by acidic stop solution. The colour changes from blue to yellow and the absorbance is measured in the photometer at 450 nm. The intensity of the yellow colour is inversely proportional to the ADMA concentration in the sample; this means high ADMA concentration in the sample reduces the concentration of tracer-bound antibodies and lowers the photometric signal. The concentration of ADMA (μmol/L) in the samples is then determined by comparing the optical density (O.D.) of the samples to the standard curve.

#### 4.5.4. Determination of Angiotensin-Converting Enzyme Concentration 

The concentration of angiotensin-converting enzyme was determined with a sandwich ELISA using Mouse Angiotensin I Converting Enzyme (ACE) ELISA Kit (Cat. No. E90004Mu was purchased from Uscn Life Science Inc., Wuhan, China). The assay was conducted according to the manufacturer’s instruction. The procedure has been described in detail in Section 4.5.2.

#### 4.5.5. Determination of Angiotensin II Concentration 

The concentration of angiotensin II was determined with Angiotension II (Human, Rat, Mouse, Porcine, Caniane) EIA Kit (Cat. No. EK-002-12 was purchased from Phoenix Pharmaceutical Inc., Karlsruhe, Germany). The assay was carried out according to the manufacturer’s instruction. The procedure has been described in detail in Section 4.5.2.

### 4.6. Statistical Analysis 

The obtained results in particular groups were given as the mean ± standard deviation (±SD), and checked for normal distribution and homogeneity of these groups. The Shapiro-Wilk test was performed for normality of the obtained results. Homogeneity of variance was evaluated with the Levene’s test with two variables. The comparison of homogenous groups and the effect of parameters (mouse model, diet type, supplement dose) on group differentiation were analysed with ANOVA and the Least Significant Differences (LSD) test.

The differences were considered to be statistically significant when a significance level was less than 0.05 (*p* ≤ 0.05). The calculations were performed with Statistica 10.0 (Polish version), and Microsoft Excel.

## 5. Conclusions

We have been the first ones to prove that bee pollen ethanol extract reduces and/or prevents the occurrence of steatosis and degenerative changes in the liver of C_57_BL_6_ mice caused by a high-fat diet, which may suggest a hepatoprotective role of EEP. This is related to a significant decrease of TC and LDL cholesterol levels, which results from including EEP into the diet. In the presented studies, when referred to our research on transgenic ApoE-knockout mice predisposed to the development of atherosclerosis, allow one to suggest that the biochemical mechanism of EEP protective effect against atherosclerosis is related to lowering ox-LDL and ADMA levels and weakening the ACE enzyme activity, which results in lowering ANG II level. Molecules of ox-LDL, ADMA, and ANG II are strongly atherogenic substances which are formed due to oxidative stress. Supplementing the diet with EEP, which has a great ability to neutralise free radicals, effectively reducing high levels of these pro-atherosclerotic substances. Noteworthy is the fact that in mice non-predisposed to the development of atherosclerosis, which were not on a high-fat diet, EEP does not exhibit negative effects and does not disturb the normal level of the analysed parameters. Further studies should be carried out to explain molecular and genetic mechanisms of bee pollen effect as well as to analyse bioavailability and biotransformation of the main polyphenol components.

## Figures and Tables

**Table 1:** Lipid profile of C_57_BL_6_ mice.

	HFD	HFD-0.1	HFD-1	SD-0.1	SD-1	SD
	High-Fat Diet (*n* = 10)	High-Fat Diet with EEP 0.1 g/kg BM (*n* = 10)	High-Fat Diet with EEP 1 g/kg BM (*n* = 10)	Standard Diet with EEP 0.1 g/kg BM (*n* = 10)	Standard Diet with EEP 1 g/kg BM (*n* = 10)	Standard Diet (*n* = 6)
TC (mg/dL)	181 ± 63 ^a^	125 ± 24 ^a,b^	118 ± 10 ^b^	73 ± 6 ^b^	67 ± 9 ^b^	69 ± 12 ^b^
LDL-cholesterol (mg/dL)	79 ± 25 ^a^	26 ± 12 ^a,b^	8 ± 4 ^b^	7 ± 2 ^b^	7 ± 3 ^b^	4 ± 2 ^b^
HDL-cholesterol (mg/dL)	68 ± 8 ^a^	68 ± 13 ^a^	78 ± 9 ^a^	40 ± 7 ^b^	36 ± 7 ^b^	40 ± 11 ^b^
TAG (mg/dL)	168 ± 52	155 ± 24	167 ± 22	129 ± 16	128 ± 22	149 ± 29

^a^ *p* < 0.05 vs. SD group; ^b^ *p* < 0.05 vs. HFD group.
